# Indents

**Published:** 1907-07-15

**Authors:** 


### INDENTS.

☞ **Brief Articles of Technical or Scientific Value will receive notice and comment. Contributions invited.**

**Deodorizer for Dental Office.** The addition of a fluidrachm of formaline to an ounce of good perfume, sprayed about your office, will keep it from smelling like a "dentist's office."—Dr. Campbell in *Summary*.

**Lower Partial Plates with Wire Connection.** In the construction of a lower partial denture I most heartily endorse the use of a wire connecting the two sides. I have been using this for years, and I find it the most satisfactory method of putting in partial plates I ever employed. —F. E. Roach in *Review*.

**Repairing Gold Fillings.** My observations have led me to place a great deal of faith in amalgam used in the patching of gold fillings which have failed, either at the cervical margin or the usual proximal margin. There is a peculiar action between gold and amalgam that renders amalgam particularly useful as a patch alongside of gold, and I believe the same holds true of tin, and for the same reason that there is a certain electro chemical action between the base metals of the amalgam or the tin and the gold filling.—W. V.-B. Ames in *Review*.

**Preserve the Root.** In operative dentistry the same importance belongs to that portion of the tooth lying below the gum as that above it, and if our work is to endure, our best efforts must be exerted to keeping the root and the tissues surrounding it in a state of health. What results do we attain even if we apply our best energies to the construction of the repair—extension for prevention, occlusal anchorage, perfect contact points, well condensed gold, finely contoured gold or porcelain crowns. When we neglect the root all may be, and frequently is, lost.—W. H. K. Moyer in *Review*.

**Investment Plaster.** Investment plaster of the most reliable and inexpensive kind can be made by any dentist from the following formula: Quantities are by measure, not by weight.

Good quality of ordinary dental plaster, 2 parts
Pulverized mica..........................................1 part
Marble dust, pulverized fine .........................1 part

This combination, if carefully and thouroughly mixed, will be found specially adapted to every department of the dentist's laboratory. While experimenting, thinking I had perfected the formula, I prematurely gave out a few samples which did not give satisfaction, but this formula will be found thoroughly reliable. The mica and marble dust should both be pulverized as fine as flour for the finest class of work. This plaster is of the quick-setting kind. The stiffer any investment plaster is mixed the quicker it will set.—H. A. Cross in *Review*.

**How to Obtain a Correct Bite** For instance take a full upper case—of course it will be the same with any other case—take impression, run model, wax up case, set on the six anterior teeth, by guess, tack on a piece of wax over ridge, where posterior teeth should be about the height the teeth should be.

Now, warm the piece of wax over ridge, try in the mouth, let patient bite down, and that will make prints of the lower teeth

You can look in the mouth and see if the patient closes naturally. Next take an impression—preferably with modeling compound—of the lower teeth, run model, then set the model in the print of the wax that was made by the lower teeth, and fasten to the articulator. The advantage over the biscuit bite is, you can see if the patient bites correctly, and it takes no longer. You will not have to keep your patient waiting over 15 to 20 minutes for you to try the case in—Dr. Lifsey in *Summary*.

**Besides Being Odorless Formidine Presents Many Advantages Over Iodoform.** It is a relief to know that an efficient substitute for iodoform has at last been discovered, and that it may be freely applied to wounds without fear of nauseating the patient and his friends with a penetrating and intensely disagreeable odor. Formidine is not a mere substitute for other iodine compounds. It will supersede iodoform because it presents advantages of its own. besides possessing a greater therapeutic efficacy than iodoform, it is a safe and powerful internal antiseptic.

Formidine is odorless, does not stain the skin or clothing, and may be easily removed, if necessary, with a little soap and water. Because of its chemical stability, Formidine lends itself to practically any form of dispensing. For internal administration it may be exhibited in powders, tablets, capsules, or with any non-alkiline substance, such as milk, coffee, chocolate, etc.

Externally, Formidine is employed as an antiseptic dusting-powder for wounds and ulcerations. Internally it is indicated in all pathological conditions of the intestinal tract depending upon infection.

It is marketed in 5-grain capsules, in one-drachm vials and one-ounce sprinkler-top bottles.

Chemically, Formidine is designated a condensation product of iodine, formaldehyde, and salycilic acid, having the formula $C_{15}H_{10}O_2I_6$ or it may be called an iodized derivative of formaldehyde and salicylic acid. It is a true chemical compound (methylene disalicylic acid iodide), exhibiting none of the physical characteristics, nor responding to any of the chemical tests for its constituent bodies until decomposed.

Formidine is a reddish-yellow powder, tasteless, and having a slight but not disagreeable odor suggestive of iodine, It is perfectly stable in the dry state, and contains about fifty per cent of iodine in loose combination. When heated, iodine is liberated, the substance melts, then chars, and is finally consumed without leaving any ash. It is insoluble in water, dilute acids, alcohol, and most other ordinary solvents. When brought in contact with alkaline secretions it slowly dissolves and develops the characteristics of its constituents, iodine, salicylic acid, and formaldehyde, and then evinces its germacidal power.

When taken internally, Formidine breaks down, as above indicated, and iodine, formaldehyde and salicylic acid are eliminated by the kidneys.

**How to Get a Fusible Metal Impression of One Tooth.**

When a dentist desires to make a fusible metal impression of a tooth he makes a plaster cast, and takes in several. It is hard to separate the individual one for which a cap is desired. To do so with a saw is slow, and to cut around the gum margin with a bur causes the bur to clog at once.

I proceed as follows: Make a plaster cap of tooth, and the adjoining teeth. Take a small piece of modeling clay—composition will do, but not nearly as well—fill the adjoining teeth to the one desired, leaving a surplus above the filled teeth. Now we have the tooth isolated. The metal is poured in, the cast held under water and knocked out of the mold, and there is the tooth desired, with wide separation from teeth not needed. There is little to be trimmed from the gum margin, and this can be done with a coarse rubber file, working between the wide space. The cap is then tried on the metal tooth, and tapped with a hammer to place. All this can be done in about ten minutes. Driving the die with cap attached against a block of lead will aid matters. The cap can be reinforced with solder, and the tooth cut in the mouth with a stone to correspond.—Dr. Spooner in *Summary.*

**The True Position of All Editors of So-called Trade Dental Journals.**

My position with respect to dental journalism is fairly well defined, even though it be considerably misunderstood. I have since 1891, that is to say, for fifteen years, been editorially directing the course of a journal which is de facto as definitely the exponent of independence in dental journalism as it is possible to produce. Independent in that its editor is endowed with practically autocratic powers to exclude from its pages anything and everything which in his judgment is obstructive to dental progress upon the best professional lines ,and free to admit everything conducive to such progress. In this spirit the editorial management of The Dental Cosmos has been carried out in the past, and so it will be conducted for the future.

It is not a commercial journal published in the interest of its Company publisher, but is issued in accordance with the motto on its cover, "in the interests of the profession," with the distinct understanding, however, that its editor is the arbiter of what constitutes the interest of the profession. I am fully aware that there may be much latitude of opinion as to "the interests of the profession." but in the editorial conduct of a dental journal unless and until its editor is clothed with something like autocratic authority to decide upon particular interests, arising under the general question of what are the best interests of the profession, no such thing as independent journalism is possible. If, instead of its editor, a committee, a board of directors, a society of the profession at large is empowered to determine what shall not be published, then independence ceases and editorial subserviency begins.

I have brought hard study and my best and fairest reasoning to bear upon this question for many years, because I love my profession, and loving it, have desired always to make my best efforts and whatever ability I may have tell in their effect by doing the best that I could for dentistry as a profession. Some few know and realize this, but many see it in only a vaulting ambition or the attempt at selfish aggrandizement, with the result that my honesty has been publicly questioned, and I have been branded as a traitor to the cause of professionalism and the creature of a grasping monopoly, that was fattening at the expense of the dental profession. It is useless to discuss the question with men holding such narrow views, and I long ago declined to do so, because, like Ephraim, they are joined to their idols. In the meantime, believing that a practical demonstration is worth more than volumes of oratory or verbal argument, I have continued my policy of concentrating my efforts in producing for the benefit of my colleagues the best dental journal that I can produce, with the resources available to me, with the result that it has succeeded beyond any previous record in its history, succeeded so that even its enemies read it, one even surreptitiously, subscribing for it in the name of the negro who attends his door bell, lest his name should appear on our subscription list. When our enemy wants The Dental Cosmos as badly as that, our friends must love and cherish it beyond measure, and it is therefore a success.

I believe in independent dental journalism in every fibre of my being, and while I have ceased to talk much about it, I am devoting my energies rather consistently to producing a typical example of journalism, which for downright, simon-pure independence is something to ponder over, and possibly a help to a rational definition of what independent dental journalism really is.—EDWARD C. KIRK, Editor *Cosmos*.

